# Supplementary material for: Oscillations of skyrmion clusters in Co/Pt multilayer nanodots
Source: Sci Rep. 2020 Oct 5;10:16517. doi: 10.1038/s41598-020-73458-6 (PMC7536206; doi:10.1038/s41598-020-73458-6)
Supplement: Supplementary file 1 [file 41598_2020_73458_MOESM1_ESM.pdf]

SUPPLEMENTARY INFORMATION

Oscillations of skyrmion clusters in Co/Pt multilayer nanodots

Felipe Tejo[1],* Felipe Velozo[1], Ricardo Gabriel Elías[1], and Juan Escrig[1,2]

[1]*Departamento de Física, Universidad de Santiago de Chile (USACH), Avda. Ecuador 3493, 917-0124 Santiago, Chile and*

[2]*Center for the Development of Nanoscience and Nanotechnology (CEDENNA), 917-0124 Santiago, Chile,*

(Dated: May 31, 2020)

* felipe.tejo@usach.cl

I. DAMPING PROCESS OF SKYRMION SIZES.

We have plotted the oscillation amplitude of the skyrmions in the time interval between 5 and 8 ns and we have calculated their trend lines. Based on the Pearson correlation coefficient R_P, we have obtained an exponential fit of the form $\omega(t) = Ae^{-bt} + C$, which confirms that the relaxation process is mediated of the skyrmion sizes follows a behavior similar to that of the damped harmonic oscillator.

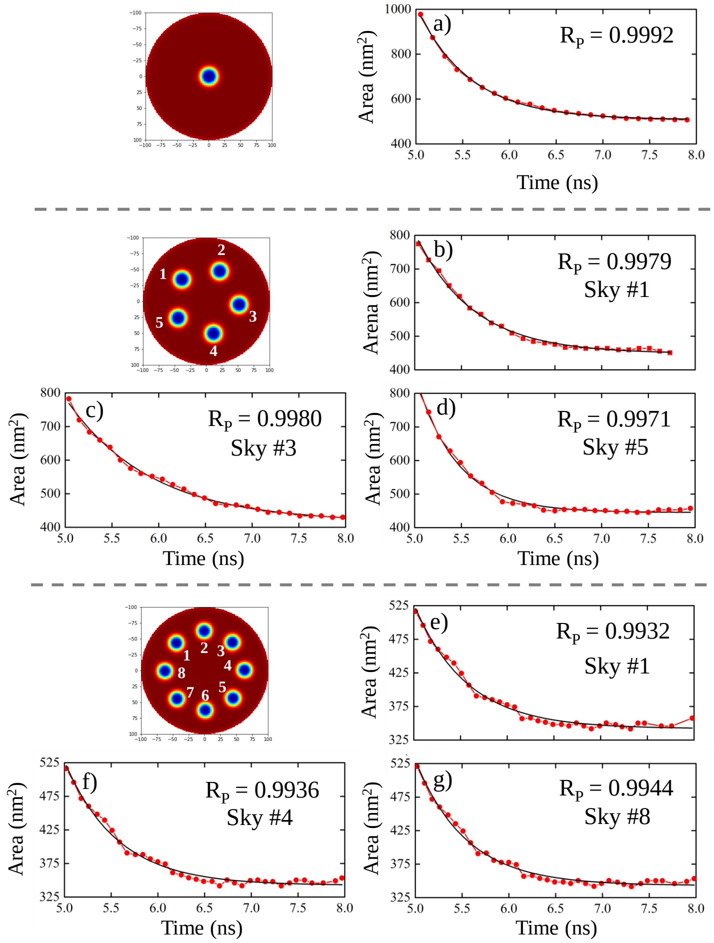

FIG. S1. Exponential fit for a) $N = 1$, b−d) $N = 5$ and e−g) $N = 8$. The symbol # identifies the skyrmion of the system represented by the snapshots. Red circles represent the peak of the skyrmion size oscillation, while the solid line corresponds to the exponential fit. R_P corresponds to the Pearson correlation coefficient, which in all cases is very close to 1.

II. LOCATION OF THE PSD PEAKS

Figure S2 shows the PSD of the oscillations when a current pulse of magnitude $J = 0.5$ A/μm^2 is applied to a cluster with $N = 5$. We found that the peaks of the oscillation frequencies of each skyrmion are positioned at a very precise point in the frequency space and are independent of the value of ε'. The above shows that the effect of ε' does not influence the natural frequency of oscillation.

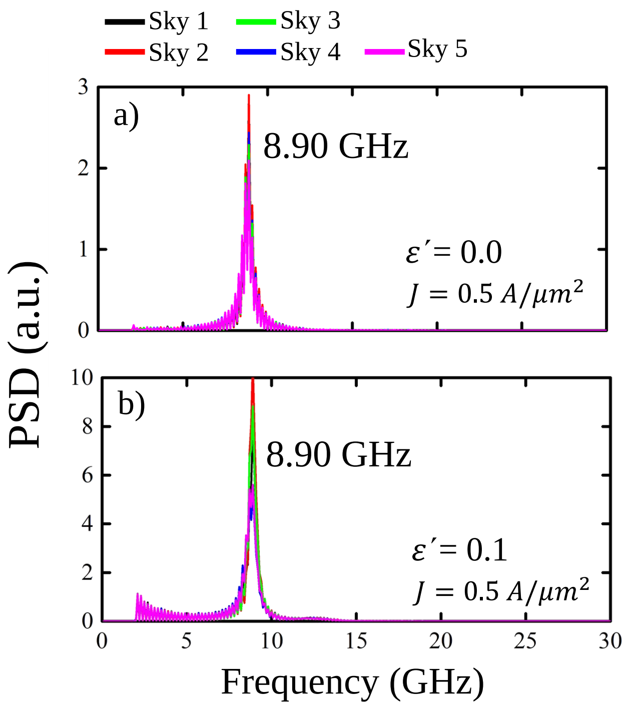

FIG. S2. Power spectral density (PSD) for a cluster of 5 skyrmions when a spin-polarized current is applied with a) ε'=0.0 (absence of field-like torque) and b) ε'=0.1. In all cases the skyrmions oscillate with the same frequency, which shows that the oscillation frequency is independent of ε'

III. RELAXATION PROCESS MEDIATED BY COHERENT OSCILLATIONS OF THE SKYRMION SIZES.

Considering the resolution used in this study, the time lags between the corresponding peaks for each individual skyrmion are really low (on the order of one hundredth of a nanometer). We have made a graph that relates the reference signal of a skyrmion with each of the other signals. From Fig. S3 we can see that the time lag is practically zero. It is important to note that for this graph we have considered between 5 and 8 ns, which is the period where the oscillation occurs.

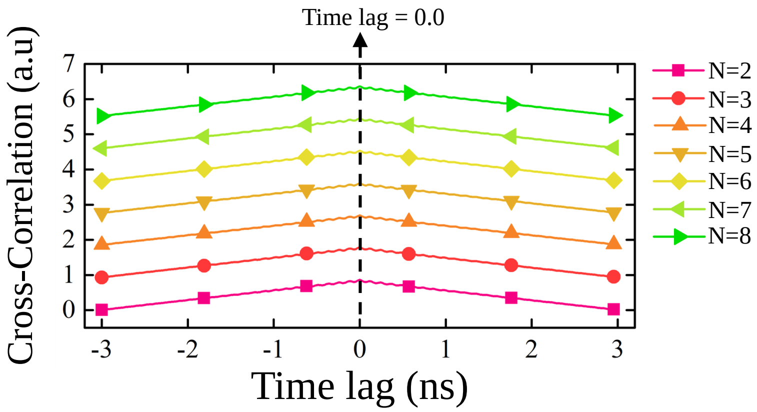

FIG. S3. Cross-correlation of the skyrmion oscillations as a function of the time lag. The peaks of the correlations are in $t = 0.0$, which shows that the skyrmions oscillate coherently. For each value of N, all curves representing the individual signals overlap.

IV. SKYRMION STABILIZATION FOR $N \geq 9$

For $N \geq 9$, the symmetry of the array is lost and the interaction between the skyrmions becomes important, causing the radius of the skyrmions to vary between the skyrmions that form a cluster. In Fig. S4, $\bar{r}$ is the average radius of the skyrmions and σ is their standard deviation. As we can see, σ is always non-zero, which means that the skyrmions in the cluster stabilize with different sizes. On the other hand, for a nanodot 200 nm in diameter, the maximum value of skyrmions is obtained for $N = 18$.

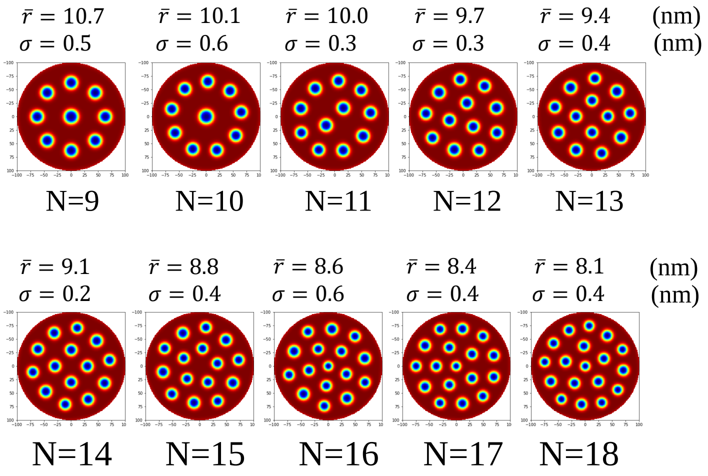

FIG. S4. Metastable states for $N \geq 9$. It can be seen that the average radius of the skyrmions $\bar{r}$ varies little with N, while standard deviation σ is always non-zero due to the different sizes of the skyrmions on the nanodot.

V. DEPENDENCE OF SKYRMION SIZE ON THE FREQUENCY OF RESPONSE.

Alternatively, as there is a close relation between skyrmion radius (r) and the number of skyrmions (N), we can also show that there is a relation between the frequency and the skyrmion radius. This relation turns to be almost linear and it can be seen in the Fig. S5.

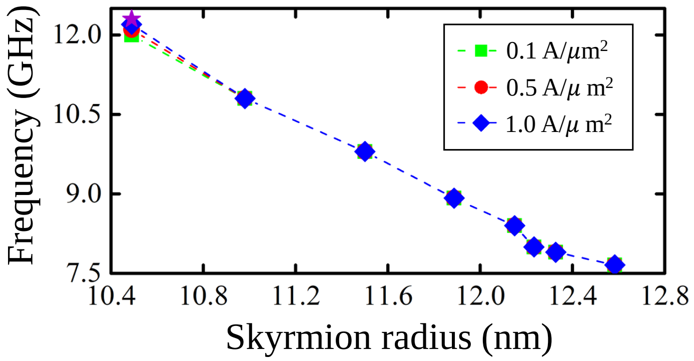

FIG. S5. Evolution of the response frequencies as a function of the skyrmion radius
